# CCL5-producing migratory dendritic cells guide CCR5^+^ monocytes into the draining lymph nodes

**DOI:** 10.1084/jem.20222129

**Published:** 2023-03-22

**Authors:** Kavita Rawat, Anita Tewari, Xin Li, Arlind B. Mara, William T. King, Sophie L. Gibbings, Chinaza F. Nnam, Fred W. Kolling, Bart N. Lambrecht, Claudia V. Jakubzick

**Affiliations:** 1 Department of Microbiology and Immunology, Geisel School of Medicine at Dartmouth, Hanover, NH, USA; 2 Dartmouth Cancer Center, Dartmouth Geisel School of Medicine, Lebanon, NH, USA; 3 Department of Pediatrics, National Jewish Health, Denver, CO, USA; 4 Laboratory of Immunoregulation and Mucosal Immunology, VIB-UGent Center for Inflammation Research, Ghent, Belgium; 5 Department of Internal Medicine and Pediatrics, Ghent University, Ghent, Belgium; 6 Department of Pulmonary Medicine, Erasmus MC, Rotterdam, Netherlands

## Abstract

Dendritic cells (DCs) and monocytes capture, transport, and present antigen to cognate T cells in the draining lymph nodes (LNs) in a CCR7-dependent manner. Since only migratory DCs express this chemokine receptor, it is unclear how monocytes reach the LN. In steady-state and following inhalation of several PAMPs, scRNA-seq identified LN mononuclear phagocytes as monocytes, resident, or migratory type 1 and type 2 conventional (c)DCs, despite the downregulation of *Xcr1*, *Clec9a*, *H2-Ab1*, *Sirpa*, and *Clec10a* transcripts on migratory cDCs. Migratory cDCs, however, upregulated *Ccr7*, *Ccl17*, *Ccl22*, and *Ccl5*. Migratory monocytes expressed *Ccr5*, a high-affinity receptor for *Ccl5*. Using two tracking methods, we observed that both CD88^hi^CD26^lo^monocytes and CD88^−^CD26^hi^ cDCs captured inhaled antigens in the lung and migrated to LNs. Antigen exposure in mixed-chimeric *Ccl5*-, *Ccr2*-, *Ccr5*-, *Ccr7*-, and *Batf3*-deficient mice demonstrated that while antigen-bearing DCs use CCR7 to reach the LN, monocytes use CCR5 to follow CCL5-secreting migratory cDCs into the LN, where they regulate DC-mediated immunity.

## Teaser

Both DCs and monocytes capture antigen for presentation to lymph node T cells. Here, Rawat et al. show that DCs use the chemokine receptor CCR7, while monocytes follow chemokines secreted by migratory DCs to reach the nodes and control immunity.

## Introduction

Antigens that are inhaled or ingested and end up in barrier mucosal tissues are not easily recognized by the adaptive immune system since they need to be taken up and transported to the draining LNs, either actively by APCs that take up the antigen and migrate to the nodes, or passively via flow in the afferent lymph to reach the LN-resident APCs that scan the LN conduit system (Vermaelen et al., 2001). Depending on the mode of transport and the degree of associated danger, the outcome of such antigen encounters can be either tolerance or immunity (Hintzen et al., 2006). Migratory conventional dendritic cells (cDCs) have a unique capacity to capture antigens across mucosal barriers and migrate to the draining LNs via afferent lymphatics; this process relies on CCR7 chemokine receptor that orchestrates lymphatic entry and LN homing to the T cell paracortex, where antigen is presented to cognate T lymphocytes (Förster et al., 1999; Hintzen et al., 2006; Plantinga et al., 2013). Not surprisingly, cell-mediated transport of inhaled and ingested soluble or particulate antigen to draining nodes is largely abolished in CCR7-deficient mice (Bakocević et al., 2010; Hintzen et al., 2006; Jakubzick et al., 2006). Yet, we and others have reported that peripheral monocytes also capture and transport antigen to LNs (Jakubzick et al., 2013; Jakubzick et al., 2017; Randolph et al., 1999), whereas these cells poorly express CCR7 on their surface. Antigen presentation by monocytes appears to be less controversial than their migratory capabilities since there are ample studies supporting monocytes as APCs when they were adoptively transferred (Coillard and Segura, 2021; Kool et al., 2008a; Kool et al., 2008b; Kool et al., 2011; Leal et al., 2021; Leon et al., 2007; Plantinga et al., 2013). Moreover, their main route of entry into the LNs is thought to be through high endothelial venules, like resident cDCs. Recently, it was found that typical monocyte-defining markers such as Ly6C and CD64 are also expressed by a subset of inflammatory cDCs, which raised concern that some studies mistook cDCs for monocytes (Bosteels et al., 2020a; Bosteels et al., 2020b; Cabeza-Cabrerizo et al., 2021; Roquilly et al., 2022). Given the lack of CCR7 expression on monocytes and the problems in correctly separating monocytes from DCs, the question remains whether CCR7 negative-to-low monocytes can indeed capture antigens in the periphery, migrate to LNs, and present antigen. To address this, we performed single-cell RNA sequencing (scRNA-seq) analysis of lung-draining LNs and found that monocytes use alternative chemotactic signals to guide their migration through afferent lymphatics in a process that requires collaboration with DCs. Using labeling techniques and mixed bone marrow (BM) chimeras, we demonstrate that monocytes enter lymphatics in a cell-intrinsic CCR5-dependent manner, dependent on a CCR7-driven migration of cDCs that produce the monocytic chemokine CCL5. Guided by this collaborative chemokine cue, monocytes shuttle antigens from the periphery to LNs and present antigens to cognate T cells for immune modulation.

## Results and discussion

### Migratory monocytes shuttle inhaled antigen to mediastinal nodes in a G protein–coupled receptor–dependent manner

**Figure 1.**
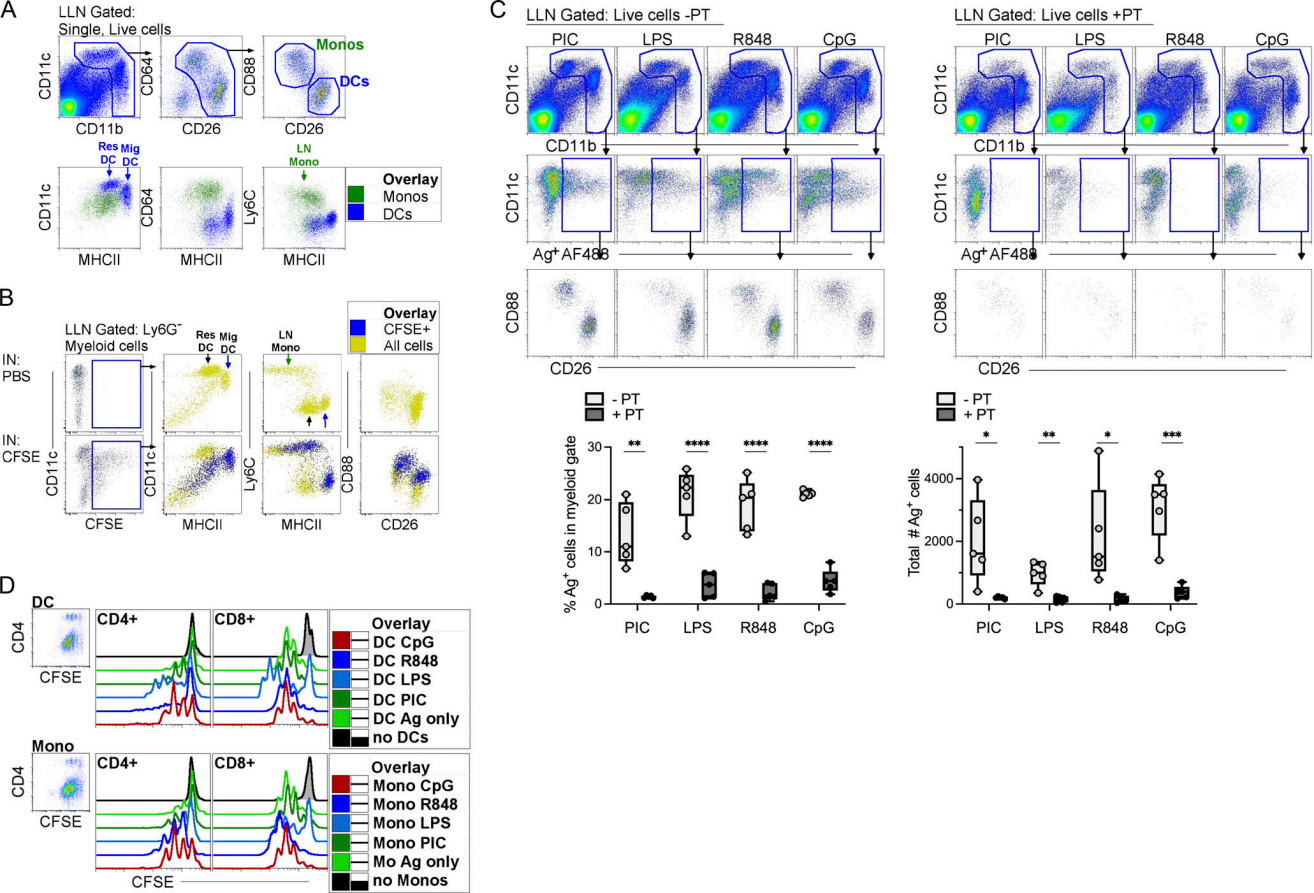
**CD88**^**+**^ **monocytes capture antigen in the periphery, transport, and present the antigen to cognate T cells in the LLNs. (A)** Top row: WT naive LLN were plotted as CD11c and CD11b to gate on myeloid cells. CD88^+^ neutrophils were excluded as CD64^−^CD26^− ^cells by plotting myeloid cells as CD64 vs. CD26. LN mononuclear phagocytes were then plotted as CD88 vs. CD26 to identify CD88^+^CD26^lo^ monocytes and CD88^−^CD26^hi^ DCs. Bottom row: Overlay of monocytes (green) and DCs (blue) to illustrate the intensity of expression of CD11c, MHCII, CD64, and Ly6C on monocytes, resident DCs, and migratory DCs. Data shown are representative of three independent experiments; *n* = 3–5. **(B)** LLN 24 h after i.n. delivery of CFSE or PBS. Top row: Control mice given PBS (no CFSE). Bottom row: Mice given CFSE. Left gate is CFSE^+^ myeloid cells. Bottom row: Overlay of CFSE^+^ migratory cells (blue) and all myeloid cells (yellow) illustrating that only migratory cDCs (MHCII high) migrate and not resident DCs (MHCII low). Data represent two independent experiments; *n* = 5. **(C)** LLN 24 h after i.n. delivery of OVA-AF488, a TLR agonist ± pertussis toxin (PT; no PT, left plots; with PT, right plots). Top row: Flow plots illustrate the myeloid gate, followed by (middle row) gated Ag^+^ myeloid cells, which were plotted as CD88 vs. CD26 to identify Ag^+^ monocytes and DCs (bottom row). Bar graphs compile the frequency and the total number of Ag^+^ myeloid cells in the LLNs; *n* = 4–5 mice per group. Data represent two independent experiments. **(D)** LLN Ag^+^ monocytes and cDCs were sorted 24 h after i.n. delivery with OVA-AF488 and a TLR agonist. Representative histograms show in vitro proliferation of CFSE-labeled antigen-specific CD8^+^ OTI and CD4^+^ OTII T cells. Data are representative of three independent experiments. *P < 0.05, **P < 0.01, ***P < 0.001, and ****P < 0.0001, mean ± SEM. One-way ordinary ANOVA, with post hoc Tukey’s multiple comparison test (C).

**Figure S1.**
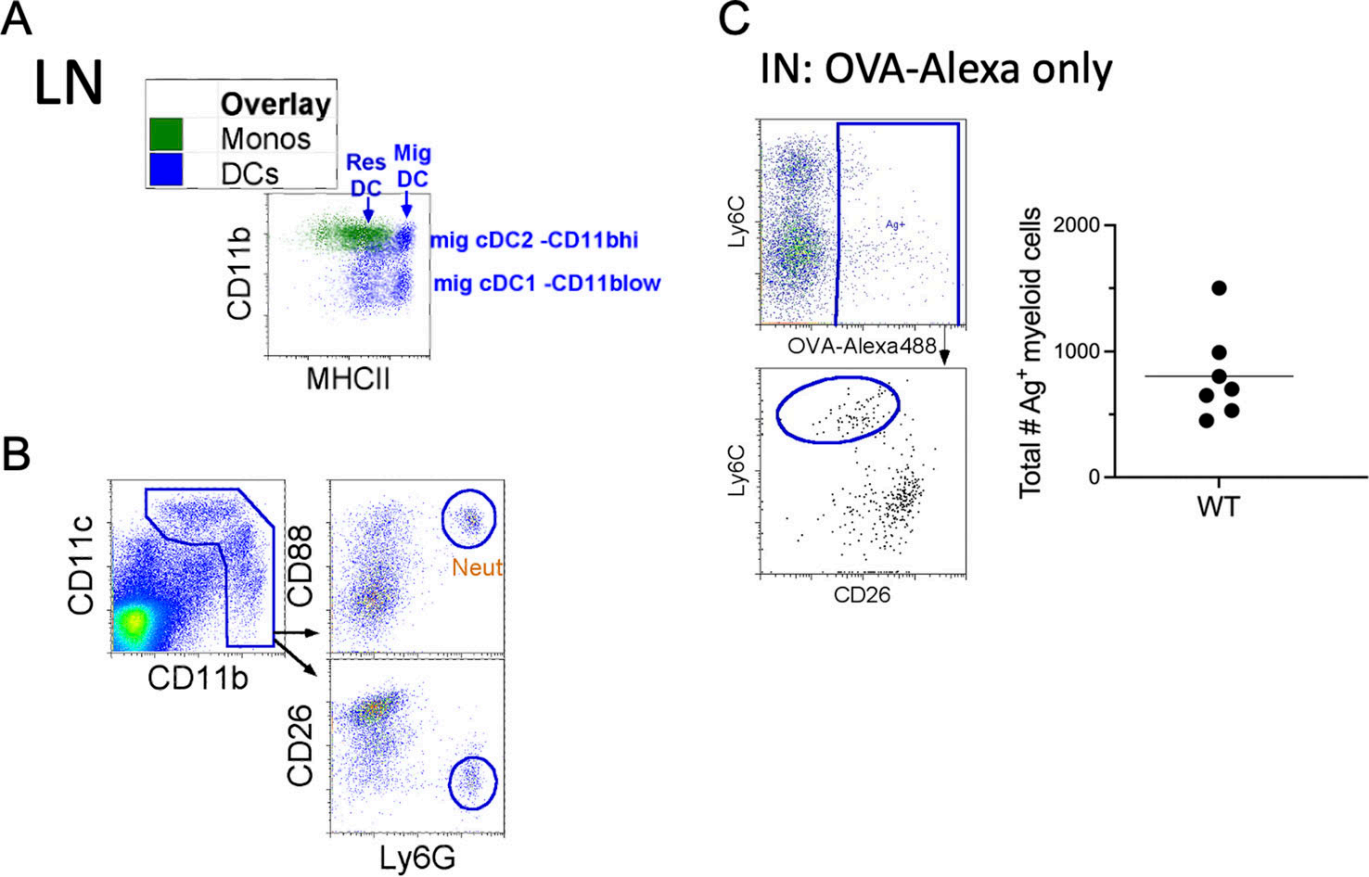
**Neutrophil exclusion and monocyte migration with OVA-AF488 without TLR adjuvants in the LLN. (A)** Overlay of LN monocytes and DCs, plotted as CD11b vs. MHCII. **(B)** Excluding CD88^+^ neutrophils from gated myeloid cells. **(C)** i.n. (IN) delivery of OVA-AF488 into WT mice, LLN harvested 24 h after instillation. Total Ag^+^ myeloid cells were assessed.

Although we and others have previously shown that Ly6C^+^ monocytes transport inhaled antigen from the lung to draining LNs in steady-state and inflammation (Jakubzick et al., 2013; Kim and Braciale, 2009; Plantinga et al., 2013), a confounder in past studies could have been the potential contamination of Ly6c^+^ cells with cDCs (Aegerter et al., 2022; Roquilly et al., 2022). To correctly identify and track the migration of antigen-bearing (Ag^+^) monocytes, we used CD88 (*C5ar*) and CD26 (*Dpp4*), along with classical myeloid cell markers CD11c, CD11b, MHCII, CD64, and Ly6C to distinguish migratory Ag^+^ monocytes from DCs (Nakano et al., 2015; Roquilly et al., 2022). After gating on myeloid cells, lung-draining LN (LLN) monocytes were CD88^hi^CD26^lo^, whereas cDCs were CD88^−^CD26^hi^ (Fig. 1 A and Fig. S1 A). Neutrophils were excluded from the analysis due to their lack of expression for CD64 and CD26; alternatively, one could exclude neutrophils using anti-Ly6G (Fig. 1 A and Fig. S1 B). Overlay FACS plots of LLN monocytes and cDCs demonstrated how resident cDCs expressed less MHCII protein than migratory DCs in steady-state (Fig. 1 A, blue cells). Compared with resident and migratory cDCs, LN monocytes had lower intensity of CD11c and MHCII staining but markedly higher CD64 and Ly6C (Fig. 1 A, green cells). Therefore, when assessing the functional role of LN APCs, a broad gate that includes high (i.e., migratory cDCs) to lower MHCII expression (resident cDCs and monocytes) is required to correctly capture all myeloid APCs.

To detect migrating cells from the lung to the LLN, mice received an i.n. instillation of CFSE, which readily labels any airway resident cells due to its spontaneous penetration of cell membranes and irreversible chemical coupling of the succinimidyl-ester-linked fluorochrome to primary amines in cellular proteins. The rapid chemical labeling of cells in vivo only labels airway resident cells and does not allow CFSE to freely enter the lymphatics and LNs (Jakubzick et al., 2008). Therefore, CFSE-labeled cells in the LLN must have migrated from the lung to the LLN. 24 h after i.n. delivery, LLN CFSE^+^ cells were migratory cDCs and monocytes, but not resident DCs, as illustrated by overlay plots in Fig. 1 B (CFSE^+^ cells blue, all myeloid cells yellow).

To address the antigen uptake and shuttling capacity of appropriately identified migratory monocytes compared with cDCs, WT mice were i.n. instilled with fluorescently labeled OVA (Ag^+^ AF488) only (Fig. S1 C) and in combination with various TLR adjuvants (i.e., Poly I:C, LPS, R848, or CpG, triggering TLR3, -4, -7, and -9) to boost APC migration and model pathogen encounter. 24 h after instillation, migratory Ag^+^ monocytes and DCs were observed in the LLN of mice given antigen with a TLR adjuvant (Fig. 1 C). To address how much of the antigen was passively entering the afferent lymph and LLN in an APC-independent manner, pertussis toxin was delivered with inhaled antigen and different TLR adjuvants to block G protein–coupled receptor (i.e., chemokine receptors) cell migration. Pertussis toxin almost completely inhibited the migration of Ag^+^ monocytes and cDCs to the LLN (Fig. 1 C), demonstrating that inhaled antigens accumulate in the LN due to specific transport by APCs that acquired the antigen in the lung barrier.

A defining characteristic of professional APCs is that antigen shuttling to LN is accompanied by the acquisition of antigen-presenting function. To address antigen presentation capacity to naive T cells, LLN Ag^+^ monocytes or cDCs were sorted from OVA-exposed mice and cultured ex vivo with cognate T cells for 4 d. In all inflammatory settings of concomitant TLR inhalation, LN Ag^+^ monocytes and cDCs induced proliferation of OVA-specific CD4^+^ and CD8^+^ antigen-specific T cells (Fig. 1 D), although cDCs induced slightly more proliferation than monocytes. These data indicate that even when carefully separated from cDCs, monocytes behave like professional APCs that capture antigens in barriers, migrate to the LLNs, and present to cognate T cells.

### Monocytes selectively express *Ccr5*, whereas migratory DCs selectively express *Ccl5*

**Figure S2.**
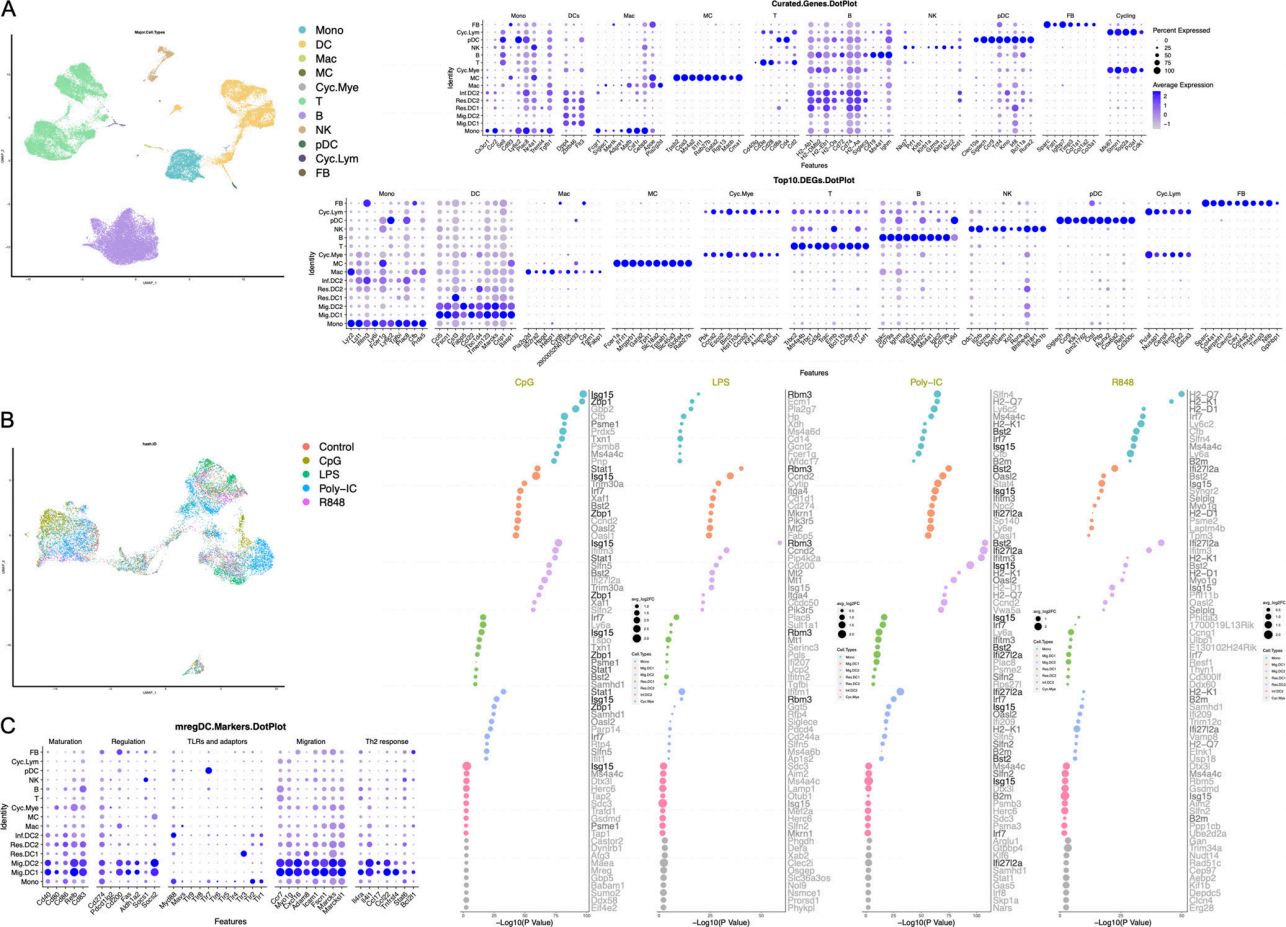
**scRNA-seq of hashtags for PBS and TLR ligand stimulated mononuclear phagocytes in the LLN. (A)** UMAP demonstrating the distribution of all cells acquired on the 10X from the five different TLR adjuvant treatment groups: no treatment control (Control), CpG, LPS, Poly-IC, R848. Dot chart shows curated genes and the top 10 DEGs for the entire dataset. **(B)** Re-clustering identified mononuclear phagocytes, UMAP demonstrating the distribution of cells from the five different TLR adjuvant treatment groups: no treatment control (Control), CpG, LPS, Poly-IC, R848. Dot chart shows the top 10 DEGs in samples with each treatment group compared with no treatment control for the seven specific myeloid cell types. UMAP annotations and platform for self-analysis: https://cells.ucsc.edu/?ds=ln-mono-dc. **(C)** Dot chart shows mregDC marker genes across defined clusters. MC, mast cells; Mono, monocytes; Cyc.Mye, cycling myeloid cells; Cyc.Lym., cyclin lymphocytes; FB, fibroblasts; NK, natural killer; pDC, plasmacytoid DC.

**Figure 2.**
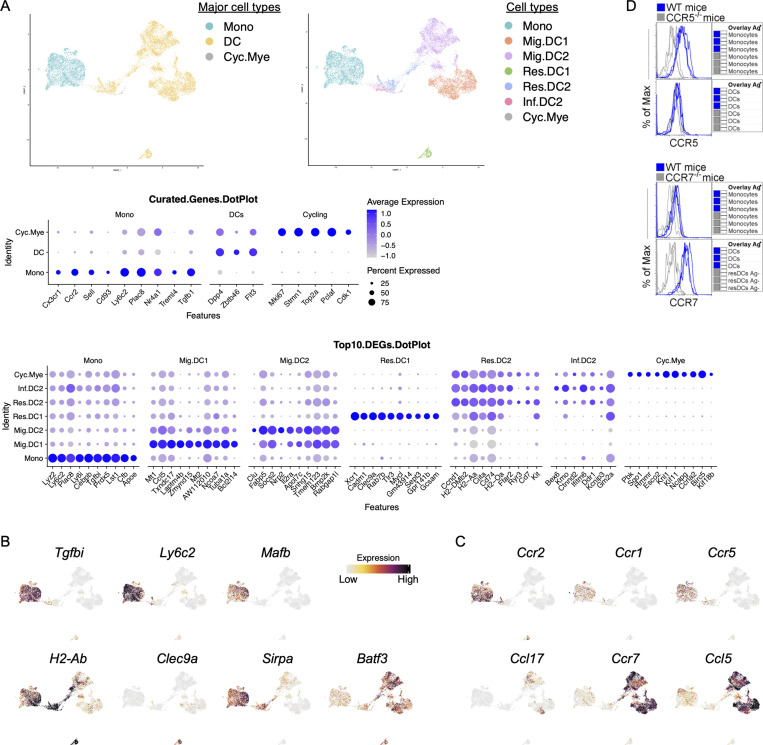
**scRNA-seq defines LN myeloid cell types with a potential internal Ccl5-Ccr5/Ccr1 axis interaction. (A)** Left: UMAP demonstrates the three major myeloid cell types: monocytes (Mono), DCs, and cycling myeloid cells (Cyc.Mye). Right: UMAP demonstrates seven distinct myeloid cell types: monocytes (Mono), migratory cDC1 (Mig.DC1), migratory cDC2 (Mig.DC2), resident cDC1 (Res.DC1), resident cDC2 (Res.DC2), inflammatory cDC2 (Inf.DC2), and cycling myeloid cells (Cyc.Mye). Middle: Dot plot shows the expression of curated genes in each individual major myeloid cell type. Bottom: Dot plot shows the top 10 DEGs in each individual myeloid cell type. **(B)** Feature plots show the expression of genes of interest including monocyte-defining *Tgfbi*, *Ly6c2*, and *Mafb;* and cDC-defining *H2-Ab*, *Clec9a*, *Sipra*, and *Batf3.* **(C)** Feature plots show the expression of genes of interest: chemokines *Ccr2*, *Ccr1*, *Ccr5*, *Ccl17*, *Ccr7*, and *Ccl5*. UMAP annotations: https://cells.ucsc.edu/?ds=ln-mono-dc. **(D)** Histograms illustrate the expression of CCR5 on monocytes (top) and CCR7 on migratory DCs (bottom). Each line represents biological replicates from three independent experiments.

To understand how antigen-presenting monocytes can reach draining LLN in the apparent absence of CCR7 expression, we performed scRNA-seq on LN mononuclear phagocytes. Sequenced cells were processed, normalized, and integrated using the Seurat package. Uniform manifold approximation and projection (UMAP) of 20 LN datasets (four for PBS-, Poly I:C-, LPS-, R848-, and CpG-treated mice) illustrated a similar cluster distribution across all samples (Fig. S2, A and B). Focusing on the myeloid cells, curated genes identified three major cell types: monocytes (*Ccr2*, *Ly6c2*, *Plac8*, *Tgfbi*, etc.), cDCs (*Dpp4*, *Zbtb46*, *Flt3*), and cycling cells (*Mki67*, *Stmn1*, *Top2a*, *Pclaf*, etc.; Fig. 2 A; Aegerter et al., 2022). Once the overarching cell types were established with curated genes, we examined the top 10 differentially expressed genes (DEGs) in an unbiased clustering analysis to define the clusters within the major cell types. The top DEGs identified seven cell types: resident cDC1 and cDC2, migratory cDC1 and cDC2, inflammatory cDCs, monocytes, and cycling myeloid cells (Fig. 2 A).

**Figure S3.**
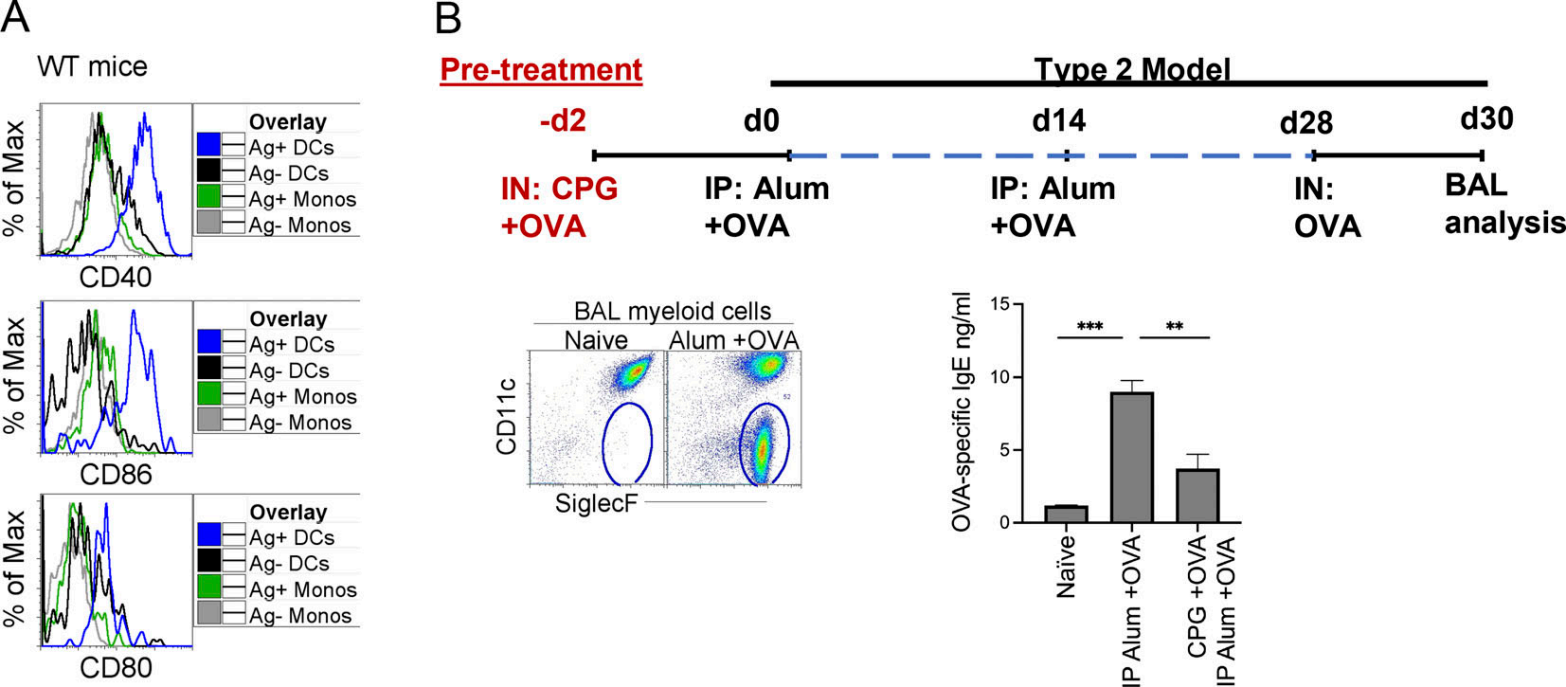
**Alum OVA model and CpG immunotherapy. (A)** 24 h after instillation of 20 μg of CpG and 3 μg OVA-AF488, LLNs were harvested and analyzed. Histograms illustrate expression of costimulatory molecules (CD40, CD80, and CD86) on Ag^+^ monocytes and migratory DCs in the LLN. **(B)** Alum OVA model. Flow plots illustrate gating strategy for eosinophils in the BAL. Graph bar, OVA-specific IgE production after alum OVA ± pretreatment with CpG and OVA. **P < 0.01, ***P < 0.001, mean ± SEM, one-way ordinary ANOVA, with post hoc Tukey’s multiple comparison test (B).

It is often assumed that protein and mRNA expression correlate; however, scRNA-seq and cellular indexing of transcriptomes and epitopes sequencing experiments have shown this is not always the case in cells of the immune system. Migratory cDCs expressed higher MHCII protein compared with resident cDCs as measured by flow cytometry (Fig. 1 A) but contained lower mRNA for *H2-Ab* (Fig. 2 B). This observation is likely due to decreased ubiquitination and MHCII recycling in migratory DCs that undergo a DC maturation process (Shin et al., 2006). Other classical genes, such as the dead cell-uptake receptor *Clec9a* are downregulated in migratory cDC1 because their expression is no longer functionally required. In fact, genes known to define cDC subsets were amply present in resident cDCs but lowly expressed in migratory cDCs, such as *Xcr1*, *Clec9a*, *Cadm1*, *Sirpa*, and *Clec10a*, most likely because migratory cDCs are in an active, terminal state no longer requiring ongoing mRNA encoding for those cell surface molecules (displaying a similar profile as previously reported for mature DCs enriched in immunoregulatory molecules [mregDCs]; Fig. S2 C and Fig. S3 A; Maier et al., 2020). The loss of commonly recognized markers such as *Xcr1* and *Sirpa* makes it difficult to differentiate between migratory cDC1 and cDC2. To address this issue, we highlight the top 10 DEGs that are specific to migratory cDC1 and cDC2 cells, such as *Mt1*, *Laptm4b*, and *Mt2* for migratory cDC1, and *Clu* and *Nrp2* for migratory cDC2, among others outlined in Fig. 2 B. Despite this reduction in key cDC-defining mRNAs, we noticed a striking gain in gene expression associated with cellular migration behavior. Migratory cDCs expressed much higher *Ccr7*, *Ccl17*, and *Ccl22.* Notably, our scRNA-seq data also revealed that *Ccl5* was highly expressed by migratory cDCs, while its cognate *Ccr5 *and* Ccr1* chemokine receptors were highly expressed by monocytes, which, as anticipated, did not express *Ccr7,* with a few expressing low amounts (Fig. 2 C). The protein expression of CCR5 and CCR7 on LN monocytes and DCs was confirmed (Fig. 2 D). These observations led us to hypothesize that CCR5-expressing monocytes require CCL5 migratory cDCs to reach the LLNs.

### Migratory cDCs employ CCL5 to recruit Ag^+^ monocytes to LNs

**Figure 3.**
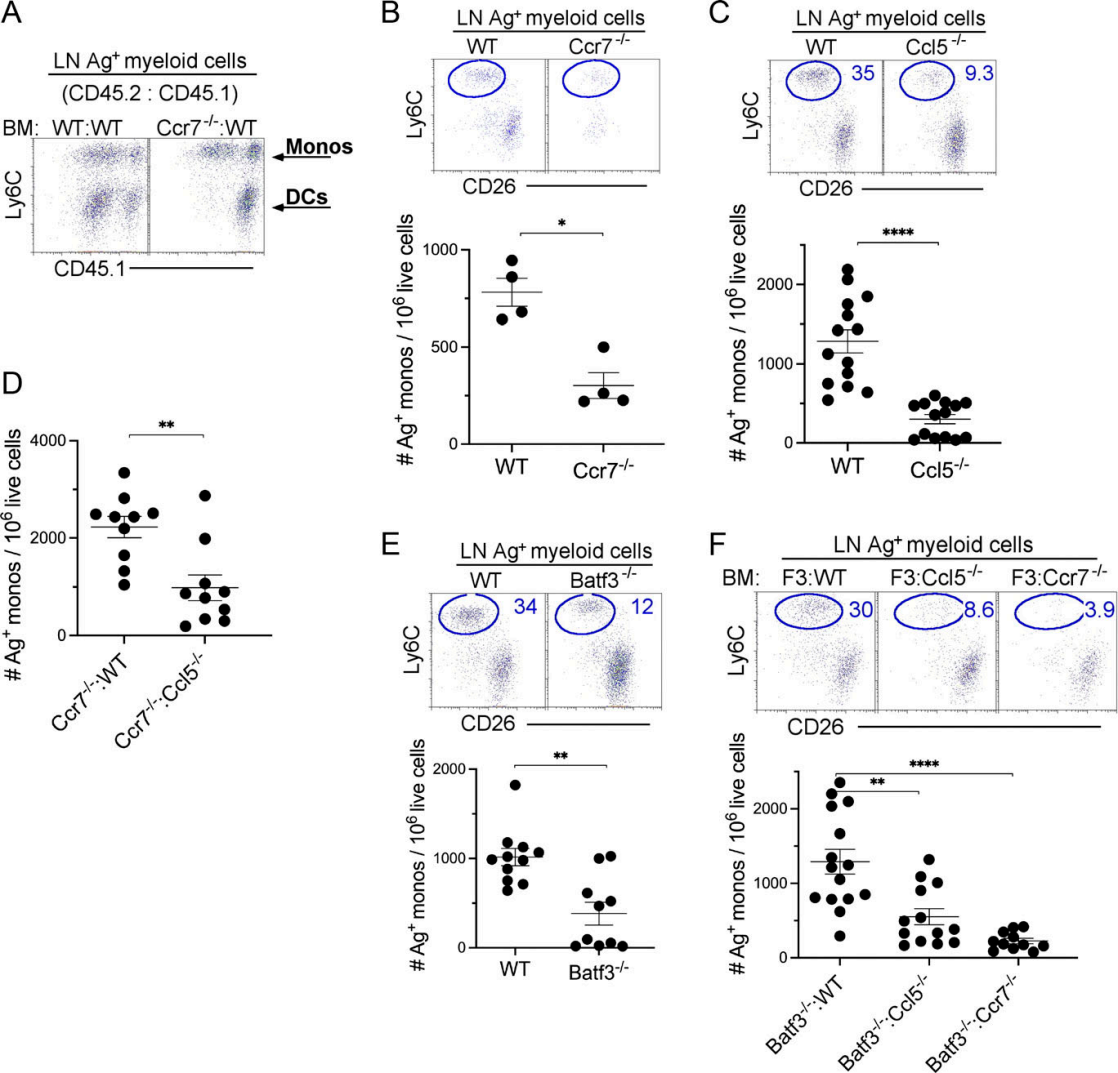
**Monocyte migration via afferent lymphatics is attenuated in the absence of CCL5-secreting migratory cDC1s.** 24 h after the instillation of 20 μg of CpG and 3 μg OVA-AF488, LLNs were harvested and analyzed. **(A)** Flow plots illustrate the Ag^+^ monocytes and DC migration in the LLN derived from (1:1) BM chimera mice: WT CD45.2:WT CD45.1 and *Ccr7*^−/−^ CD45.2:WT CD45.1 mice. **(B)** Representative flow plot with scatter plot showing the total number of Ag^+^ monocytes in WT and *Ccr7*^−/−^ mice with scatter plot showing the total number of Ag^+^ monocytes. Data are representative of two independent experiments; *n* = 4 per group. **(C)** Representative flow plot with scatter plot showing the total number of Ag^+^ monocytes in WT and Ccl5^−/−^ mice with scatter plot showing the total number of Ag^+^ monocytes. Data combine three independent experiments; *n* = 4–5 per group. **(D)** Scatter plot showing the total number of Ag^+^ monocytes in *Ccr7*^−/−^: WT CD45.1 and *Ccr7*^−/−^: *Ccl5*^−/−^ (1:1) BM chimera mice. Data combine two independent experiments with four to five mice per group. **(E)** Representative flow plot with scatter plot showing the total number of Ag^+^ monocytes in WT and *Batf3*^−/−^ mice with scatter plot showing the total number of Ag^+^ monocytes. Data combine two independent experiments with five mice per group. **(F)** Representative flow plot with scatter plot showing the total number of Ag^+^ monocytes in *Batf3*^−/−^:WT, *Batf3*^−/−^:*Ccl5*^−/−^, and *Batf3*^−/−^:*Ccr7*^−/−^ BM chimera mice. Each dot represents one mouse. *P < 0.05, **P < 0.01, ****P < 0.0001, mean ± SEM, a two-tailed *t* test (B–E) and one-way ordinary ANOVA, with post hoc Tukey’s multiple comparison test (F).

While CCR7 is not expressed by LLN Ag^+^ monocytes, it was still important to functionally test if CCR7 is required for monocyte migration to LNs, particularly since earlier experiments had shown that most cell-dependent antigen transport in the lungs depends on *Ccr7* (Plantinga et al., 2013). To examine this, we created 1:1 (CD45.2 *Ccr7*^−/−^:CD45.1*Ccr7*^+/+^) BM congenic chimeric mice in which *Ccr7*^−/−^ cells competed with *Ccr7*^+/+^ WT cells. Control BM chimeras addressing the confounding effect of the congenic locus containing multiple gene polymorphisms (CD45.2:CD45.1 WT:WT) were set up in parallel. After reconstitution, these chimeric mice received inhalation of fluorescently labeled OVA in the presence of CpG adjuvant, and migration to the LLN was measured 24 h later. Whereas in WT:WT mice, Ag^+^ monocytes and DCs in the LLN derived equally well from CD45.2^+^ and CD45.1^+^ donor cells (Fig. 3 A), in *Ccr7*^*−/−*^*:Ccr7*^*+/+*^ mice, only CCR7-sufficient antigen-carrying cDCs were observed in draining LN, confirming the key role for CCR7 in cDC migration to the nodes. Strikingly, however, CCR7-deficient CD45.2 antigen-carrying Ly6C^+^ monocytes were equally capable of reaching the draining LN as CCR7-sufficient monocytes, showing this to be a CCR7-independent process for monocytes (Fig. 3 A).

Since the migration of cDCs from the tissue to the draining LNs depended on cell-intrinsic expression of CCR7, whereas that of monocytes did not, we next studied monocyte transport in full *Ccr7*^−/−^ mice. As shown in Fig. 3 B, total deficiency of CCR7 reduced the accumulation of Ag-carrying monocytes in the LLN, suggesting that cDC migration is a prerequisite for monocyte migration to the LLN, and that the loss of monocyte Ag transport in *Ccr7*-deficient mice is a cell-extrinsic effect.

Given that monocyte migration required cDC migration to the nodes, we next examined the mechanism by which cDCs recruited monocytes and focused on CCL5, made so abundantly by migratory cDCs, and where its high-affinity receptor, CCR5, is expressed on monocytes. To address this, WT and *Ccl5*^−/−^ mice were instilled with fluorescently labeled antigen and CpG. Compared with WT mice, *Ccl5*^−/−^ mice displayed ∼75% reduction in Ag^*+*^ monocytes reaching the LLN, whereas Ag^+^ cDC migration to the LN was unaffected (Fig. 3 C). To further demonstrate that migratory CCR7^+^ cDCs recruited monocytes to LNs in a CCL5-dependent manner, we created a different set of BM chimeric mice where all the migratory cDCs (i.e., cDC1 and cDC2) were either CCL5 sufficient (*Ccr7*^*−/−*^*:*WT mice) or CCL5 deficient (*Ccr7*^−/−^:*Ccl5*^*−/−*^ mice). In *Ccr7*^*−/−*^*:Ccl5*^*−/−*^ mice, we observed ∼77% reduction in Ag^+^ monocytes migrating to the LLNs compared with *Ccr7*^*−/−*^*:*WT mice (Fig. 3 D), strongly suggesting that CCL5 secretion by migratory cDCs is a critical chemokine for monocyte recruitment to the LNs.

Since CCL5 is prominently expressed by migratory cDC1s, we next investigated whether the cell-intrinsic absence of *Ccl5* in cDC1s reduced the migration of Ag^+^ monocytes to the LLN. We first found that the complete absence of cDC1 in *Batf3*^−/−^ mice resulted in an ∼60% reduction in Ag^+^ monocytes migrating to the LLNs, approaching the severity of the migration defect seen in *Ccl5*^−/−^ mice. This result suggests that monocyte migration is dependent on cDC1 migration and while cDC2 can partially contribute, it cannot fully compensate for the loss of cDC1 (Fig. 3 E). Next, we reconstituted *Batf3*^−/−^ mice with either CCL5-sufficient DC1 or CCL5-deficient DC1 by setting up chimeric *Batf3*^−/−^:*Ccl5*^+/+^ mice and *Batf3*^−/−^:*Ccl5*^−/−^ mice to confirm the lack of monocyte migration in the *Batf3*^*−/−*^ mice was due to the lack of expression of CCL5 and not due to other aspects of the DC itself. In addition, we also created a mouse in which only cDC1 migration was defective by creating chimeric *Batf3*^−/−^:*Ccr7*^−/−^ mice. Both CCL5- and CCR7-deficient cDC1 mice demonstrated a significant reduction in monocyte migration compared with mice where cDC1 were CCL5- and CCR7-sufficient (Fig. 3 F). These data suggest that CCL5 secretion by cDC1s is critical for monocyte recruitment and migration into draining LN in the context of concomitant CpG exposure.

### Monocyte migration to LNs depends on cell-intrinsic CCR5

**Figure 4.**
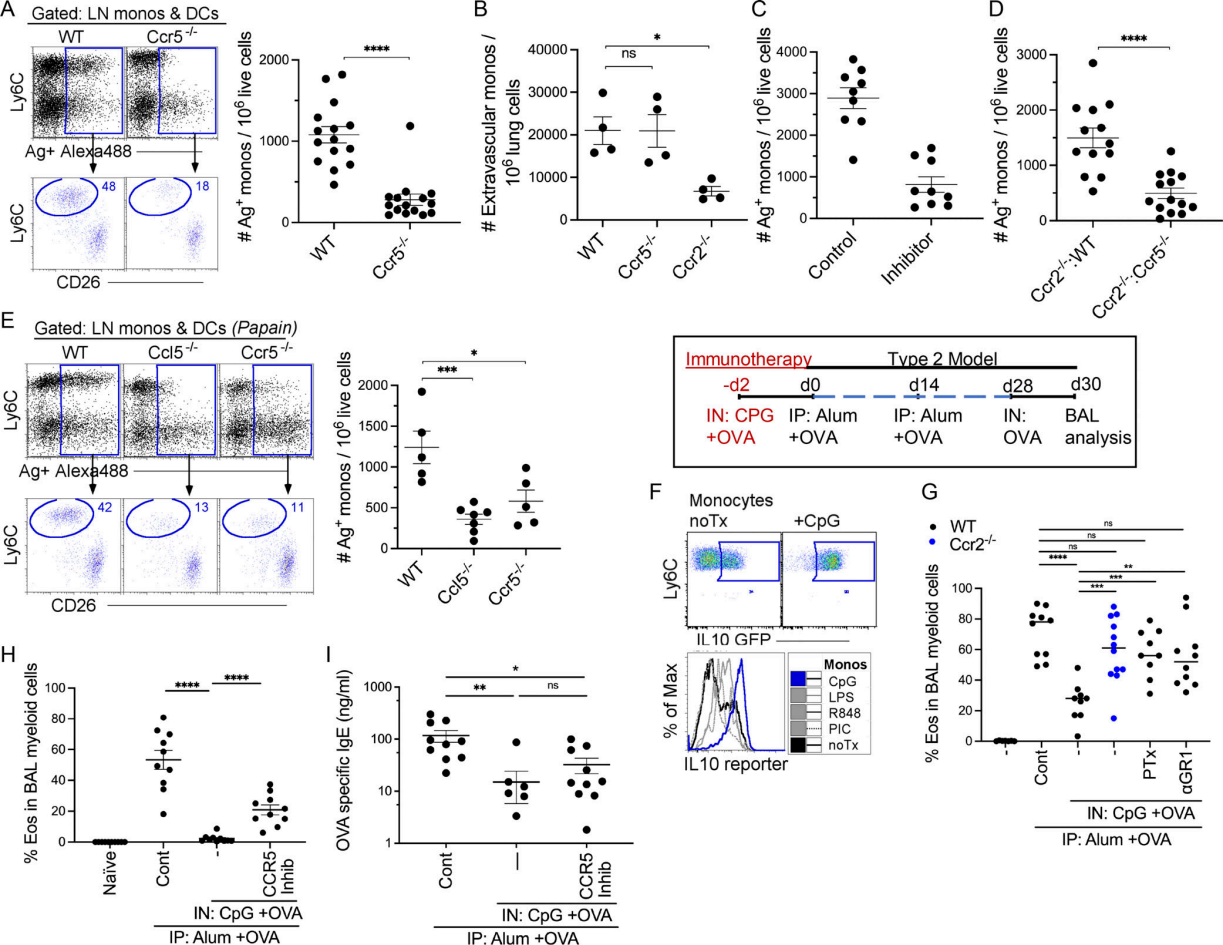
**Monocyte migration to draining LNs depends on CCR5 expression. (A)** 24 h after instillation of 20 μg of CpG and 3 μg OVA-AF488, LLNs were harvested and analyzed in WT and *Ccr5*^−/−^ mice. Flow plots, myeloid cells plotted as Ly6C vs. OVA^+^AF488^+^ to gate on Ag^+^ monocytes and DCs with scatter plot showing the total number of Ag^+^ monocytes in WT and *Ccr5*^−/−^ mice. Data combine two independent experiments; *n* = 4–5 per group. **(B)** WT, *Ccr5*^−/−^, and *Ccr2*^−/−^ mice were analyzed for extravascular monocyte migration into the lungs after immunization. Data represent two independent experiments. Each dot represents one mouse. **(C)** WT mice were treated with 20 μg Maraviroc (CCR5 inhibitor) 4 h prior to i.n. delivery with 20 μg of CpG and 3 μg OVA-AF488. Scatter plot displays the number of Ag^+^ monocytes in the LLN. Data combine two independent experiments with four to five mice per group. **(D)** Scatter plot illustrates the number of Ag^+^ monocytes in the LLNs of *Ccr2*^−/−^:WT and *Ccr2*^−/−^*:Ccr5*^−/−^ BM chimeric mice. Data combine three independent experiments with four to five mice per group. **(E)** WT, *Ccl5*^−/−^, and *Ccr5*^−/−^ mice were i.n. instilled with 8 μg of papain and 3 μg OVA-AF488 and harvested 24 h later. Flow plots illustrate myeloid cells plotted as Ly6C vs. OVA^+^AF488^+^ to gate on Ag^+^ monocytes and DCs with scatter plots showing the total number of Ag^+^ monocytes. Data represent two independent experiments with three to four mice per group. **(F)** Flow plot shows IL10 expression of LN monocytes 24 h after CpG stimulation. Histogram monocyte overlays illustrate IL10 reporter expression of monocytes stimulated with different TLR agonists. **(G)** Control mice received no CpG-OVA prior to sensitization with Alum + OVA and challenge with OVA. *Ccr2*^−/−^ mice, WT mice with blocked APC migration (pertussis toxin [PTx]) or depleted of monocytes (anti-Gr1 antibody) during exposure to CpG-OVA, developed significantly more airway eosinophilia compared to CpG-OVA treated WT mice. Scatter plot shows the frequency of eosinophil migration into the airways. Data combine three independent experiments with three to five mice per group. **(H)** Scatter plot shows the frequency of eosinophil migration into the airways of WT mice treated with CpG-OVA ± CCR5 inhibitor (Maraviroc) followed by sensitization with alum + OVA and OVA challenge. Data combine two independent experiments with four to five mice per group. **(I)** Scatter plot illustrates the total OVA-specific IgE in the serum of the mice from experiment H. *P < 0.05, **P < 0.01, ***P < 0.001, ****P < 0.0001, mean ± SEM, a two-tailed *t* test (A–D), and one-way ordinary ANOVA, with post hoc Tukey’s multiple comparison test (E–I).

The chemokine receptor CCR5 expressed on monocytes is a high-affinity receptor for CCL5, but monocytes also express CCR1. We, therefore, examined whether CCR5 was required. Compared with WT mice, *Ccr5*^−/−^ mice displayed a ∼75% reduction in Ag^+^ monocyte migration to the LLN when OVA and CpG were given to the mice (Fig. 4 A). Importantly, this reduction was not due to the absence of monocyte entry into peripheral tissues as observed in *Ccr2*^*−/−*^ mice that have defective monocyte egress from the BM and entry into tissue (Fig. 4 B; Serbina et al., 2003). It was notable that the remaining Ag^+^ monocytes present in the *Ccr5*^−/−^ mice only carried low amounts of Ag (top flow panels, Fig. 4 A), suggesting that these might be resident LN monocytes that acquired the antigen from LN conduits and Ag^+^ DCs upon entry into the LN. To further assess monocyte migration dependency on CCR5, we used a synthetic CCR5 antagonist (Maraviroc) in vivo and observed a ∼70% reduction in Ag^+^ monocytes in the LLN compared with vehicle control mice (Fig. 4 C). Next, we created BM chimeric mice where 100% of the monocytes were either CCR5 sufficient (*Ccr2*^−/−^:*Ccr5*^+/+^) or CCR5 deficient (*Ccr2*^−/−^:*Ccr5*^−/−^) to examine whether the requirement for CCR5 on monocytes was cell intrinsic. Indeed, CCR5-deficient monocytes were less competent to migrate to LLN than their CCR5-sufficient counterparts (Fig. 4 D). Lastly, we also observed that the non-TLR adjuvant papain induced CCR5-dependent migration of Ag^+^ monocyte to the LLN (Fig. 4 E). Collectively, the data demonstrate that monocytes require mainly cell-intrinsic CCR5 expression for peripheral antigen transport to the nodes.

### Migratory LN monocytes dampen type 2 immunity

Monocytes express immunosuppressive mediators such as Tgfb and IL10. Previous studies from our lab demonstrated that IL10-secreting monocytes exert a regulatory effect on a cDC–Poly I:C–induced cytotoxic T cell response (Tewari et al., 2021). Since monocytes in this setting function to regulate immunity, we first examined the expression level of IL10 using an IL10 reporter mouse and showed that regardless of the TLR adjuvant given, monocytes increased their expression of IL10. However, CpG elicited the highest production of IL10 (Fig. 4 F). Since prior exposure to antigen and inhaled CpG is known to regulate type 2 immunity in an airway allergic model, either due to an IL-10–dependent suppression or due to induction of a counterregulatory Th1 response (Sabatel et al., 2017; Shirota et al., 2000), we hypothesized that exposure to CpG-OVA would result in Ag^+^ monocytes regulating OVA-induced type 2 immune response. WT mice sensitized with alum-OVA, followed by an i.n. challenge with OVA antigen, displayed significantly increased airway eosinophilia and production of OVA-specific IgE antibodies compared with naive controls. In WT mice pretreated with CpG-OVA, there was a significant reduction in airway eosinophilia and IgE antibody production (Fig. 4 G and Fig. S3 B). However, if monocytes were absent (as addressed in *Ccr2*^*−/−*^ mice), their migration blocked (by in vivo use of inhaled pertussis toxin), or depleted (using anti-Gr1 antibody) during the CpG-OVA treatment, mice developed significantly more airway eosinophilia compared with CpG-OVA treated WT mice (Fig. 4 G). Blocking monocyte migration using a CCR5 inhibitor during the CpG-OVA treatment resulted in a similar observation (Fig. 4, H and I). This suggests that CpG elicits immunoregulatory migratory monocytes that have the potential to suppress type 2 immunity in the lungs through mechanisms that remain to be further explored.

Monocytes have often been seen as progenitors to monocyte-derived DCs (moDCs) with clear antigen-presenting capacity in vitro and in vivo*.* This has formed the basis of many clinical trials in cancer patients (Romero et al., 2016). Moreover, monocyte transfer in mice lacking cDCs has been able to restore key antigen-presenting functions, pointing to a clear potential of monocytes as APCs (Coillard and Segura, 2021; Kool et al., 2008b; Kool et al., 2011; Leal et al., 2021; Leon et al., 2007; Plantinga et al., 2013). Nevertheless, there have been some recent doubts showing that the protocols used to generate mouse moDCs, mainly generate macrophage-like cells and cDC2s (Helft et al., 2015) and that the classical way by which moDCs were defined in vivo by flow cytometry, were essentially often a contamination of cDC2s and macrophages (Bosteels et al., 2020b). When those macrophages were separated from the cDC2 contaminants from the lungs, essentially all APC potential within the moDC fraction disappeared. This is because monocytes can rapidly transit through tissues without necessarily differentiating into moDCs or macrophages (Jakubzick et al., 2013; Jakubzick et al., 2017; Randolph et al., 1999).

In this study, we show that the true potential of monocytes as APCs is only revealed in the draining nodes, where these cells disguise as resident cDCs, displaying only moderate amounts of MHCII and CD11c, and therefore are often missed as migratory APCs. Although monocytes share many cell surface markers with inflammatory DCs and macrophages, they can be reliably distinguished using cell surface markers CD88 (*C5ar1*), CD26 (*Dpp4*), and Ly6C (*Ly6c2*) for functional studies (Nakano et al., 2015). Like cDCs, monocytes capture antigens in the periphery and migrate to the draining LNs. However, the mechanism used to enter lymphatic vessels are distinct. While cDCs require cell-intrinsic CCR7 for afferent lymphatic migration, monocytes require cell-intrinsic CCR5. Strikingly, migratory cDCs collaborate with monocytes and orchestrate their migration by producing CCL5, explaining why the absence of migratory cDCs in CCR7-deficient mice was always reported to eliminate all cell-mediated Ag transport to the nodes, as we also observed here. Our study shows that the selective absence of cDC1 migration results in a marked reduction of monocyte migration to the draining LNs. These findings suggest that, despite expressing CCL5, cDC2s may not contribute as significantly to monocyte migration in response to the immunostimulants used. It is possible that other stimuli, such as allergens or helminth-derived products, which activate cDC2s more effectively, might lead to increased expression of CCL5 by migratory cDC2 and could therefore, in this context, play a more prominent role in monocyte migration to LNs compared to CpG-induced inflammation. An alternative explanation could be that *Batf3* not only controls cDC1 development but also the production of chemokines in cDC2s or chemokine receptors on monocytes, which would also hamper monocyte migration in *Batf3*^−/−^ mice. Such a scenario is possible given that Batf3 expression is upregulated in migratory cDC2s.

Since monocytes and cDC both cargo antigen to the draining LN, what might be the unique function of monocytes? Previously we showed that monocytes downregulate the induction of a Poly I:C cDC1-mediated immune response via IL10 production and the induction of suppressor CD4^+^ T cells (Tewari et al., 2021), and here, we now report that monocytes can dampen a type 2 immune response when exposed to CpG, a mechanism also reported previously for interstitial macrophages (Sabatel et al., 2017). However, there are instances where monocytes have a stimulatory function, such as when they are stimulated by a TLR7 agonist and become cross-presenting APCs that induce an antigen-specific CTL response (Larson et al., 2016). In other instances, they seem to produce IL-12 in trans, helping the priming of Th1 responses induced by cDCs in the setting of CpG adjuvant exposure, another explanation as to why CpG motifs could suppress type 2 immunity in the lung (De Koker et al., 2017; Leal et al., 2021; Shirota et al., 2000). A monocyte–cDC collaborative model is very likely to occur when migratory cDCs express CCL5 chemokine that recruits CCR5-expressing monocytes with them as they migrate to the T cell area. Future studies will have to address if a DC-derived trail is responsible for the recruitment of CCR5 monocytes and if it already starts in the periphery to drag monocytes into crossing lymphatic endothelial cells or only sets in as these cells arrive in the nodes. In support of the former, we did observe in other experiments that Ccl5 also marks mature *Ccr7*^+^ cDCs in the periphery. Overall, in every immune response there are accelerators and brakes, and these need to work in a collaborative way, when and where it matters. Our data show that monocytes should be seen as fully competent members of a group of migratory professional APCs whose functional migration to the LNs ultimately depends on DCs, and depending on the context, there are times when they may promote or resolve immunity.

## Materials and methods

### Mice

C57BL/6 Ly5.1 (CD45.1) and Ly5.2 (CD45.2), WT mice were purchased from Charles River/NCI or Jackson Laboratory. B6.129P2-Ccl5tm1Hso/J, B6.129P2-Ccr5tm1Kuz/J, B6.129S4-Ccr2tm1Ifc/J, B6.129S(C)-Batf3tm1Kmm/J, B6.129P2(C)-Ccr7tm1Rfor/J, B6.129S6-Il10tm1Flv/J, OTI, and OTII were purchased from Jackson Laboratory. All mice were genotyped or phenotyped prior to studies and used at 6–12 wk of age, housed in a specific pathogen–free environment at Dartmouth Hitchcock Medical College, an Association for Assessment and Accreditation of Laboratory Animal Care–accredited institution, and used in accordance with protocols approved by the Institutional Animal Care and Utilization Committee. Both genders were used with no gender-specific effects noticed.

### BM chimeras

8-wk-old Ly5.1 (CD45.1) WT mice were lethally irradiated with two doses of 460 rad 12 h apart. After the second irradiation, recipient mice received 5 × 10^6^ donor BM cells i.v. comprising 80:20 of the following BM genotypes: *Batf3*^−/−^:WT CD45.1, *Batf3*^−/−^:*Ccl5*^−/−^, *Batf3*^−/−^:*Ccr7*^−/−^, *Ccr2*^−/−^:WT CD45.1, *Ccr2*^−/−^:*Ccr5*^−/−^, and 1:1 mixtures of WT CD45.2:WT CD45.1, *Ccr7*^−/−^:WT CD45.1, and *Ccr7*^−/−^:*Ccl5*^−/−^. Mice were analyzed at least 8 wk after reconstitution. In *Batf3*^−/−^:WT CD45.1, *Batf3*^−/−^:*Ccl5*^−/−^, and *Batf3*^−/−^:*Ccr7*^−/−^ BM chimeras, the DC1 niche was 100% reconstituted with either WT CD45.1, Ccl5^−/−^ or Ccr7^−/−^ DC1; all other radiosensitive cells were derived from 80% *Batf3*^−/−^ BM. In *Ccr2*^−/−^:WT CD45.1 and *Ccr2*^−/−^:*Ccr5*^−/−^, the monocytes niche available was 100% reconstituted with either WT CD45.1 and *Ccr5*^−/−^ BM, all other radiosensitive cells were derived from 80% *Ccr2*^−/−^ BM.

### Flow cytometry and single-cell suspensions

The LLNs were teased with 26 G needles and digested in 1 ml of 2.5  mg/ml collagenase D (Roche) solution in 1X RPMI at 37°C for 30 min. Digestion was stopped with 100 μl EDTA (100 mM). The cells were homogenized with glass Pasteur pipettes and then filtered through a 70-μm nylon filter. Single-cell suspensions were collected and centrifuged at 300 *g* for 5 min.

mAbs and isotype-matched control mAbs purchased from BioLegend were used for flow cytometry staining: PE-conjugated to CD26, Vα2, and SiglecF; PerCP-Cy5.5–conjugated to CD64, XCR1, and CD4; PE-Cy7–conjugated to CD11c and CD45.1; BUV395-conjugated to CD11b; BUV805-conjugated to CD8a, FITC-conjugated to F4/80, Ly6C, and CD103; allophycocyanin-conjugated to CD88 and CD64; APC-Cy7–conjugated to Ly6C, Ly6G, CD45, and CD11c; BV421-conjugated with Ly6G; and BV510-conjugated to MHCII and CD45.2. The viability dye DAPI (#D9542; Sigma-Aldrich) was added immediately before each sample acquisition on a BD Symphony A3 analyzer (BD Biosciences). Data were analyzed using FlowJo (Tree Star). Antigen-specific antibodies and isotype controls were obtained from BioLegend, eBioscience, and BD Biosciences.

### Immunization

Mice were immunized i.n. with 40 μl of 3 μg OVA-AF488 (#034781; Invitrogen) combined with individual treatments with following different TLR adjuvants: 2 μg LPS (#L8274; Sigma-Aldrich), 50 μg Poly I:C (#ALX-746-021; Enzo), 50 μg R848 (#ALX-420-038; Enzo), 20 μg CpG/ODN1668 (#ALX-746-051; Enzo), and non-TLR adjuvant: 8 μg Papain (#76216; Sigma-Aldrich). i.n. immunization with CFSE alone was performed at a concentration of 5 μM in 40 μl. WT mice were immunized with 3 μg OVA-AF488 combined with TLR adjuvant ± pertussis toxin (2 μg/mouse). After 24 h, LLNs were harvested and analyzed for Ag^+^-positive myeloid cells.

### In vitro T cell proliferation

24 h after immunization with TLR ligands combined with OVA, Ag^+^ monocytes and DCs from mediastinal LNs were sorted (FACS Aria Fusion, BD). CFSE-labeled OTI and OTII cells were cocultured with Ag^+^ monocytes or Ag^+^ DCs for 4 d in RPMI with 10% FCS, 1% Pen/Strep/L-glutamine (Sigma-Aldrich), 1% non-essential amino acids (Sigma-Aldrich), 1% sodium pyruvate (Sigma-Aldrich), 10 mM Hepes (Sigma-Aldrich), and 0.1 mM β-mercaptoethanol containing 10 μM OVA peptides (OVA^257-264^ and OVA^323-339^). Flow cytometric analysis was performed to examine the proliferation of OTI and OTII cells.

### CCR5 inhibitor study

WT mice were i.p. injected with 20 μg of Maraviroc (14641; Cayman). 4 h later, the mice were immunized i.n. with 40 μl of 3 μg OVA-AF488 and 20 μg of CpG. After 24 h, LLNs were harvested and analyzed for Ag^+^-positive myeloid cells.

### Lung analysis for extravascular monocytes

Mice were immunized i.n. with 40 μl of 10 μg OVA and 20 μg of CpG. 24 h later, the mice were injected with anti-CD45 antibody to exclude intravascular cells from the analysis. The lungs were perfused with cold 1× PBS, minced, and digested with 2.5 mg/ml collagenase D solution for 30 min at 37°C. 100 μl of 100 mM EDTA was added to stop 1 ml of enzymatic digestion. Digested tissue was pipetted up and down 30 times using a glass Pasteur pipette and passed through a 70-μm nylon filter to acquire single-cell suspensions.

### Induction of allergic airway inflammation with OVA-alum

Two groups of mice: the first group, the experimental group, was given i.n. 40 μl of 20 μg CpG and 10 μg OVA (Sigma-Aldrich) on 2 d prior to sensitization. The control group and the experimental group were both sensitized with two doses of 20 μg OVA adsorbed to 500 μg alum hydrogel (Invivogen) i.p. in 300 μl 1× PBS at days 0 and 14. Mice were challenged i.n. with 20 μg OVA on day 28. On day 30, mice were sacrificed, and different parameters were analyzed. i.p. injection with CCR5 inhibitor (Maraviroc) was performed at d0 (−4 h) followed by CpG + OVA i.n. instillation. To determine the Th2 cell–dependent eosinophilic airway inflammation in bronchoalveolar lavage (BAL) fluid, lungs were lavaged with 4 ml saline, BAL cells were counted, and eosinophils were analyzed and counted. Serum was extracted to measure circulating OVA-specific IgE (RnD systems).

### scRNA-seq analysis

scRNA-seq data are available at GEO under accession number GSE215299.

### Mouse treatment

Mice were immunized as described before without TLR adjuvant or with individual different TLR adjuvants: Poly I:C, LPS, CpG, and R848 in 40 μl sterile PBS 24 h before harvest. The LLNs were harvested, and single-cell suspensions were prepared. Cells were stained with flow cytometry antibodies and different TotalSeq A antibodies (BioLegend) according to the different TLR adjuvant treatments. After the staining, single-cell suspensions were washed three times by resuspension in 4.5 ml HBSS plus 0.5% BSA, followed by centrifugation at 300 *g* for 5 min at 4°C. After the final wash, cells from different treatment groups were resuspended and mixed. Myeloid cells were sorted as shown in Fig. S2 with the exclusion of Ly6G neutrophils. Sorted cells were centrifugated at 300 *g* for 5 min at 4°C. Supernatants were aspirated and pellets were resuspended with HBSS plus 0.5% BSA at an approximate concentration of 2.5 × 10^5^ cells/ml. Cell quality and viability were assessed with a Cellometer K2 (Nexcelom Bioscience). All samples had viability >35%. Single cells were then processed using the Chromium Next GEM Single Cell 3′ Platform (10X Genomics). Approximately 60,000 cells were loaded on each channel with an average recovery rate of 27,000 cells. Libraries were sequenced on NextSeq2000 (Illumina) with an average sequencing depth of 40,000 reads/cell.

### Data preparation

Raw sequencing reads were demultiplexed, mapped to the GRCm38 mouse reference genome, and gene expression matrices were generated using CellRanger v6.1 (10X Genomics). The following analyses were conducted in R 4.1. Seurat package v4.0 was used for downstream data analyses, and figures were produced using the package ggplot2. Following a standard workflow, the gene expression matrix was filtered to discard cells with less than 200 genes, as well as genes that were expressed in <3 cells. Samples then underwent quality control to remove cells with either too many or too few expressed genes (average around 1,500 and 4,300) and cells with too many mitochondrial RNA (average around 7.5%), resulting in a total of 43,412 cells. At the same time, sample demultiplexing and identification of cell treatment were accomplished in R using Seurat “HTODemux” function based on the filtered feature-barcode matrix generated by CellRanger. Then, “SCTransform” was applied with the “glmGamPoi” method to normalize gene expression data. After individual preparation, all the samples were introduced into a combined Seurat object via “FindIntegrationAnchors” and “IntegrateData” functions. Then, the scaled values of variable genes were subject to principal component analysis for linear dimension reduction. A shared nearest neighbor network was created based on Euclidean distances between cells in multidimensional principal component (PC) space (the first 50 PC were used) and a fixed number of neighbors per cell (50 neighbors). This was used to generate a two-dimensional UMAP for visualization as a harmonized atlas to dissect the cell populations from different treatment groups.

### DEGs

DEGs were calculated with “FindAllMarkers” function of Seurat in R 4.1 to study the different expression profiles in different cell types and upregulated genes in different treatment groups. The “data” matrices of “SCT” assay were used, and the minimal log fold change was set to 0.25. Only genes that were detected in >25% of cells in either of the two populations were used to compute the DEGs with the Wilcoxon rank-sum test. Markers were identified as genes exhibiting significant upregulation when compared against all other clusters and defined by having a Bonferroni-adjusted P value <0.05. The DEGs are ranked by the adjusted P value to select the top DEGs for downstream analysis.

### Cell type identification

To identify cell types, the “FindClusters” function with the Leiden algorithm with various resolutions from 0.5 to 2.0 in the Seurat package was used for clustering. The “FindAllMarkers” function was then applied. The top DEGs of individual clusters were examined for well-studied marker genes across literature, and the clusters were then annotated for the most likely identity. 11 distinct cell types were identified, including monocytes, DCs, cycling myeloid cells, macrophages, mast cells, T cells, B cells, natural killer cells, plasmacytoid DCs, cycling lymphocytes, and fibroblasts (not shown). Monocytes, DCs, and cycling myeloid cells were isolated and reclustered for the purpose of investigating their regulatory role during immune responses. “FindClusters” was performed with different resolutions until the right resolution is reached so that each cluster has unique gene expression pattern. Again, the “FindAllMarkers” function was then applied, and the top DEGs were examined to annotate the clusters. Migratory DC1, migratory DC2, resident DC1, resident DC2, and inflammatory DC2 were eventually identified and included in the final scRNA-seq layout. UMAP annotations and platform for self-analysis: https://cells.ucsc.edu/?ds=ln-mono-dc.

### Statistical analysis

Statistical analyses were performed using GraphPad Prism (GraphPad Software). All results are expressed as the mean ± SEM; dots represent individual measurements.

Data were analyzed using ordinary one-way ANOVA (normal distribution) with Tukey’s post hoc multiple comparison test when comparing more than two groups and two-tailed Student’s *t* test when comparing two groups, respectively. Data containing more than two groups and two independent variables were analyzed with a two-way ANOVA with Sidak’s post hoc test for multiple comparisons.

### Online supplemental material

Fig. S1, A and B, demonstrate the overlay of monocytes and DCs in the LLNs and the gating strategy for the exclusion of CD88^+^ neutrophils, respectively, and are associated with Fig. 1 A. Fig. S1 C illustrates the migration of OVA-AF488^+^ (no TLR ligand) monocytes and DCs during steady-state, which was not included in Fig. 1 C. Fig. S2 A shows UMAP, which illustrates all the cells acquired on 10X from control and TLR ligand treatments, and a dot chart that shows curated genes with the top 10 DEGs related to Fig. 2. In Fig. S2 B, the UMAP shows the reclustering of identified mononuclear phagocytes in the control and treatment groups, and the dot chart displays the top 10 DEGs for the specific myeloid cell types, respectively, and is related to Fig. 2. Fig. S2 C shows marker genes for mregDCs not shown in Fig. 2. Fig. S3 A shows the expression levels of co-stimulatory molecules CD40, CD86, and CD80 on the migratory (Ag^+^ and Ag^−^) DCs and monocytes in the LLNs and is related to Fig. 2. Fig. S3 B includes the experimental layout for the alum-OVA–induced airway allergic disease model, gating strategy for eosinophilia, and total OVA-specific IgE production using ELISA for the experiments done in Fig. 4, G–I.

## Data Availability

The data underlying Figs. 1, 2, 3, and 4 are available in the published article and its online supplemental material. The data underlying Fig. 2, A–C, are openly available at https://cells.ucsc.edu/?ds=ln-mono-dc. scRNA-seq data are available at GEO under accession number GSE215299.
